# Association between Adiposity and Iron Status in Women of Reproductive Age: Data from the UK National Diet and Nutrition Survey (NDNS) 2008–2019

**DOI:** 10.1016/j.tjnut.2024.08.026

**Published:** 2024-09-03

**Authors:** Sabrina P Demirdjian, Maeve A Kerr, Maria S Mulhern, Paul D Thompson, Mark Ledwidge, Mary T McCann

**Affiliations:** 1 Nutrition Innovation Centre for Food and Health (NICHE), School of Biomedical Sciences, Ulster University, Coleraine, Northern Ireland; 2 School of Medicine, University College Dublin, Ireland

**Keywords:** iron deficiency, anemia, central adiposity, obesity, overweight, childbearing, hemoglobin, transferrin receptor, inflammation, hematological markers

## Abstract

**Background:**

Overweight/obesity and iron deficiency (ID) are highly prevalent in women of reproductive age (WRA), impacting on women’s health. Obesity is a risk factor for nutritional deficiencies but its association with ID is unclear.

**Objectives:**

To determine the association between adiposity and markers of iron status and ID prevalence in WRA.

**Methods:**

This cross-sectional study analyzed the National Diet and Nutrition Survey (2008–2019) data, focusing on women aged 18–49 y with body mass index (BMI) ≥18.5 kg/m^2^. Prevalence of anemia, iron deficiency anemia (IDA), and ID were analyzed. Ferritin was adjusted for C-reactive protein. Iron status was assessed across high and low BMI, waist circumference (WC), waist-to-height ratio (WHtR), and waist-to-hip ratio (WHR). χ^2^, linear and logistic regressions were performed adjusting for covariates.

**Results:**

Among 1098 WRA, 496 normal weight and 602 overweight/obesity, prevalence rates were: anemia 9.2% and IDA 6.8%. Anemia was more prevalent in those with higher WHtR and WHR (11.9% compared with 5.9% and 16.7% compared with 6.5%, both *P* < 0.001). WRA with increased WC, WHtR, and WHR had higher IDA prevalence than those with lower adiposity (8.5% compared with 4.3%, *P* = 0.005; 9.4% compared with 3.3%, *P* < 0.001; 12.1% compared with 4.9%, *P* < 0.001). ID prevalence was 49.7% (ferritin cutoff 30 μg/L) and 19.6% (ferritin cutoff 15 μg/L), showing similar rates across adiposity groups. ID prevalence defined by soluble transferrin receptor (sTfR) was higher in women with increased WHR (*P* = 0.001). Higher WHR predicted ID categorized by sTfR (adjusted odds ratio [aOR]: 2.104, *P* = 0.004), and WHtR and WHR predicted anemia and IDA (anemia: WHtR aOR: 2.006, *P* = 0.036; WHR aOR: 4.489, *P* < 0.001 and IDA: WHtR aOR: 2.942, *P* = 0.012; WHR aOR: 4.142, *P* < 0.001).

**Conclusions:**

At least 1 in 5 WRA in the UK are iron deficient, highlighting the need to revise current policies. Greater central adiposity was strongly associated with impaired iron status and the development of anemia, IDA, and ID.

## Introduction

Overweight/obesity represents the most common chronic condition reported in women of reproductive age (WRA) with severe implications for women’s health. With a prevalence of 60% in UK WRA and a persistent upward trend [1], this increased adiposity can result in early onset disability that includes cardiovascular disease, type 2 diabetes, stroke, cancer, liver/gall bladder disease, osteoarthritis, chronic kidney disease, and gynecological problems [2]. Likewise, weight stigma and discrimination favor poor self-esteem, anxiety, and depression [2] and in those who become pregnant, obesity is associated with increased maternal and perinatal mortality [3].

Iron deficiency (ID) is a common global concern, with WRA particularly at risk. Although differences exist globally in precise diagnosis, ID is generally defined as low iron stores with low ferritin concentrations. When ID is severe enough to reduce hemoglobin (Hb) concentrations, ID anemia (IDA) is diagnosed. WRA have higher iron requirements than the general population [4], with these requirements becoming significantly higher in pregnancy [5]. Despite this, daily iron intake in this population is significantly lower than the recommended [6], placing WRA in a state of nutritional vulnerability.

ID in WRA is a serious public health issue globally, not only in low- and middle-income countries but also in high-income regions, where the prevalence of anemia is 16% in WRA [7] and affects 1 in 4 in the UK [8]. This deficiency can lead to serious adverse consequences including palpitations, headache, dizziness, restless legs, fatigue, irritability, anxiety, and depression [9]. IDA in pregnancy increases maternal mortality and morbidity, raising the incidence of obstetric complications, including postpartum hemorrhage, preterm birth, and stillbirth [10,11].

The association between adiposity and iron status is yet to be fully elucidated. It has been hypothesized that WRA with overweight/obesity may be at increased risk of ID because of reduced iron absorption. In obesity, iron absorption has been reported to be approximately two-thirds of the absorption in women of normal weight [12]. Iron absorption has been inversely associated with blood ferritin levels, increasing when ferritin concentrations are low and decreasing when they are high [13,14]. Alongside possible reduced iron absorption, some inflammatory markers are reported to be increased in obesity including C-reactive protein (CRP), IL-6, and ferritin [[15], [16], [17], [18], [19], [20]]. Hormonal regulation of iron is maintained by the protein hepcidin [21], with elevated concentrations in situations of iron overload and decreased concentrations during episodes of hypoxia or ID [22,23]. It has also been suggested that hepcidin levels could be inappropriately high during obesity, but studies so far have yielded conflicting results [[24], [25], [26], [27], [28], [29]]. Although no differences in Hb have been demonstrated [30], lower transferrin saturation (TSAT) [12,19,24,31] and higher soluble transferrin receptor (sTfR) concentrations have been found in obesity compared with normal weight [16,20], suggesting that adiposity could be a risk factor for the development of ID.

Identifying ID in WRA with obesity is notably challenging, leading to potential misdiagnoses and impeding the evaluation of its prevalence and treatment. In the UK, clinical guidelines do not recommend routine screening for ID or prophylactic iron supplementation in WRA, with ferritin testing advised only for those with specific symptoms or risk factors [32,33]. As obesity is associated with the presence of chronic inflammation [34], ferritin, which is an acute phase reactive protein, may increase in the presence of inflammation [20,35]. This falsely high ferritin could potentially mask a possible ID, called functional ID [36] putting these women at a greater risk of missed diagnosis. Therefore, risk and prevalence of ID development in WRA with greater adiposity is currently unknown and needs to be urgently addressed.

This study aims to determine the association between adiposity and a range of markers of iron status and ID prevalence in WRA using nationally representative data from the UK National Diet and Nutrition Survey (NDNS) 2008–2019.

## Methods

The STROBE checklist for cross-sectional studies was followed.

### Study design, setting, and participants

#### The NDNS

The NDNS is a UK cross-sectional survey that aims to obtain high-quality national quantitative data on diet, nutrient intake, and nutritional status of the general population, to help inform and develop policies and monitor progress toward the objectives set by the UK Department of Health. Detailed methodology can be found in the NDNS user guide (https://doc.ukdataservice.ac.uk/) and previous reports [37]. The program began in 1992, focusing on children (1.5–4.5 y) and adults over 65 y of age; since 2008, it has included individuals aged 18 mo or older. The current analysis used data collected during the first (2008/2009–2011/2012, years 1–4), second (2012/2013–2013/2014, years 5–6), third (2014/2015–2015/2016, years 7–8), and fourth periods (2016/2017–2018/2019, years 9–11).

#### Sampling and survey stages

Using random sampling based on private addresses across the UK, 1 adult was randomly selected from each household. The first stage consisted of a face-to-face interview termed Computer-Assisted Personal Interview (CAPI) in which general information was collected and a 4-d food diary was given to each participant. Height and weight measurements were measured at this time. Those participants who completed CAPI and ≥3 d of the 4-d food diary were then invited to the second stage of the survey.

The second stage consisted of a visit by a qualified nurse within 2–4 mo after the first stage. A fasting blood sample was taken, additional anthropometric measurements were performed such as waist circumference (WC) and hip circumference (HC), and information regarding prescribed medicines and dietary supplements were collected.

#### Participants

**FIGURE 1:**
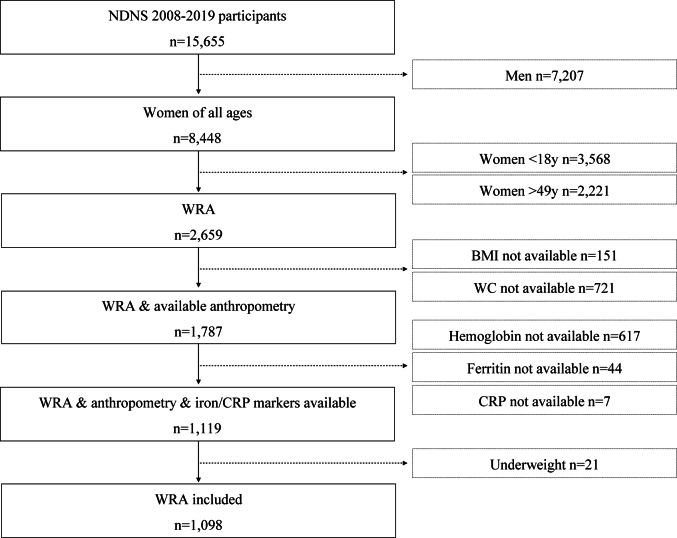
Flowchart of the steps followed to select the sample population. Available anthropometry, BMI and WC; available iron markers, hemoglobin (g/L), and ferritin (μg/L); CRP, C-reactive protein; NDNS, National Diet and Nutrition Survey (2008–2019, years 1–11); WC, waist circumference; WRA, women of reproductive age.

For the purpose of the current analysis, only nonlactating and nonpregnant WRA were included (excluding men, women aged <18 or >49 y). Those participants who did not have anthropometric measurements, Hb, ferritin, or CRP results were excluded (Figure 1). In addition, women with BMI <18.5 kg/m^2^ were excluded.

#### Anthropometric measurements

For all anthropometric measurements, participants were required to be lightly dressed, barefoot and heavy jewelry removed. For weight and height measurements, participants were standing facing forward, with feet together and arms at the sides of the body and according to the Frankfort plane. Soehnle, Seca 850, Seca 870, and Tanita THD-305 scales were used for weight measurements. BMI was calculated using the calculation kg/m^2^. For the WC measurement, the waist was defined at the midpoint between the iliac crest and the lower costal margin. For HC measurement, the hip was defined as the widest circumference above the gluteal muscles and below the iliac crest. An insertion tape calibrated in mm with a metal buckle at one end was used.

#### Biochemical measurements

A fasting blood sample was taken from all consenting participants. A complete blood count was performed using a Beckman Coulter LH700 series analyzer and for the 9–11 y Siemens Advia 2120, employing the Coulter Principle, for red blood cells, white blood cells, platelets count, as well as mean cell volume (MCV) measurement. Mean cell hemoglobin (MCH) and red cell distribution width (RDW) were derived from these measurements on the basis of standard clinical calculations (MCH = Hb/red blood cell count, RDW = (standard deviation of MCV/MCV) × 100) [38,39]. Hb was measured using the cyanmethemoglobin method. This involved spectrophotometric measurement at 525 nm in a sample diluted 1:256 with an isotonic diluent and lysing solution. The lysing agent destroyed the red cells, releasing hemoglobin into the solution and allowing white blood cell count estimation via the Coulter Principle without red cell interference. The lysing reagent also converted hemoglobin to cyanmethemoglobin. Complete cell count was performed in the Department of Hematology and the Department of Clinical Biochemistry and Immunology at Addenbrooke’s Hospital.

The method used for measuring plasma ferritin was immunonephelometry on the Dade Behring ProSpec in the first 5 y (first period and the first year of the second period) and was changed to immunoturbidimetry on the Siemens Dimension Xpand in years 6–10. In year 11, ferritin analysis was performed on serum using the Siemens Dimension EXL200 analyzer at the Core Biochemical Assay Laboratory (CBAL).

For the analysis of sTfR, the enzyme immunoassay method was used in years 1–5, based on the double antibody sandwich method (Ramco Laboratories Inc.). The evaluation of this marker was discontinued in the fifth year.

High-sensitivity CRP analysis was conducted at the Elsie Widdowson Medical Research Council Laboratory (MRC EWL), using the Dimension RXL at Addenbrooke’s for years 1–5, which changed to Dimension Xpand at MRC EWL for years 6–10, detecting CRP concentration from 1.0 mg/L. In year 11, this analysis was performed at the CBAL, on a Siemens Dimension EXL200 analyzer, extending its detection range from 0.5 to 250 mg/L.

Quality control was assessed both internally and externally for all markers. If results did not meet standards, analysis was stopped to ensure only results within quality control parameters were reported. Internal quality control for full cell count data was not available for reporting, but results were validated automatically. Representative laboratory assays were CV% were 3.1% (red blood cells (RBC)), 1.5% (MCV), 3.1% (MCH), 2.4% (RDW), and 1.8% (Hb). Externally, results were assessed by the UK National External Quality Assessment Service (UKNEQAS) and compared with the All Laboratories Trimmed Mean, which is specific to laboratories using the same analyzer and method as Addenbrooke’s. NEQAS provided a “performance index” based on how the laboratory’s results deviated from the consensus mean. Scores above 100 indicated unacceptable performance, 80–100 indicated borderline performance, and below 80 indicated acceptable performance.

Control serum with low, medium, and high ferritin concentrations were used in each run. The results were verified to ensure they fell within the manufacturer’s target range. External quality assessment was through the NEQAS Haematinics scheme.

Detailed data on quality controls and assessment can be found in the NDNS appendixes [40, 41]

#### Dietary intakes

Participants recorded food intake over 4 consecutive days (2 weekdays/2 weekend days) using a paper diary. Portion sizes were estimated using household measurements, labeled weights, and photographs of the most frequently consumed foods. Where possible, participants were asked to record the brand names to aid with nutritional information of unusual foods. Food intake was entered into a modified version of MRC Human Nutrition Research dietary assessment system (Diet In Nutrients Out, DINO) in Microsoft Access. For data on food composition, the Department of Health’s NDNS Nutrient Databank was used, incorporated into the DINO system. When portions were described as small, medium, and large, the weight was recorded on the basis of the Food Standards Agency [42].

All participants included in this analysis completed ≥3 of the 4 d of the food diary. Nutrient intake per day and per 10 MJ of energy intake was calculated both from the diet and with the inclusion of vitamin supplements. The percentage of misreporting energy intake was calculated using the following formula: Energy intake – Estimated energy requirements/Estimated energy requirements) × 100. Nutrients intake were adjusted for the percentage of misreporting energy needs.

#### Definitions

Anemia was defined as Hb <120 g/L. ID was defined using 2 markers: ferritin and sTfR concentrations. We considered 3 definitions of ID: ferritin <30 μg/L [32], ferritin <15 μg/L [35], and sTfR ≥5.81 mg/L [43]. IDA was defined as Hb <120 g/L and ferritin <30 μg/L [32] or <15 μg/L [35]. Ferritin was adjusted for inflammation by performing an internal regression correction as previously described [35,44,45].

Participants were classified into 2 groups on the basis of their BMI, WC, waist-to-height ratio (WHtR), and waist-to-hip ratio (WHR) (Table 1). According to BMI, participants were classified into Normal weight: BMI 18.5–24.9 kg/m^2^ or Overweight/Obesity: BMI: ≥25 kg/m^2^ [46]. According to WC, participants were classified into WC <80 cm or ≥80 cm [47]. According to WHtR, participants were classified into WHtR <0.50 or ≥0.50 [46]; and using WHR, participants were classified into WHR <0.85 or ≥0.85 [47].

**TABLE 1:** Classification of low and high adiposity using BMI, WC, WHtR, and WHR.

Adiposity measure	Low adiposity	High adiposity
BMI^1^ (kg/m^2^)	18.5–24.9	≥25
	*n* = 496	*n* = 602
WC^2^ (cm)	<80	≥80
	*n* = 410	*n* = 688
WHtR^1^	<0.50	≥0.50
	*n* = 440	*n* = 658
WHR^2^	<0.85	≥0.85
	*n* = 751	*n* = 347

Abbreviations: WC, waist circumference; WHR, waist-to-hip ratio; WHtR, Waist-to-height ratio.

Unweighted number of participants included per adiposity category.

1 NICE guideline, 2023.

2 World Health Organization. Waist circumference and waist-hip ratio: report of a WHO expert consultation, Geneva, 2008.

### Statistical analysis

Normality was checked using the Kolmogorov–Smirnov test. Continuous variables were described with mean and standard deviation and categorical variables with absolute frequency and percentage.

The prevalence of anemia, IDA (Hb <120 g/L and ferritin cutoff 30 or 15 μg/L), and ID (ferritin cutoff 30 and 15 μg/L; sTfR ≥5.81 mg/L) was compared between adiposity groups using χ^2^ test.

Markers of iron status and CRP were considered dependent variables including Hb, ferritin, RDW, MCH, MCV, mean cell hemoglobin concentration (MCHC), sTfR, as well as ID and IDA. Measures of adiposity (BMI, WC, HC, WHtR, and WHR) were considered independent variables.

Linear regression analysis was performed between measures of adiposity as continuous variables (weight, BMI, WC, HC, WHtR, and WHR) and markers of iron status and inflammation (Hb, RBC, MCH, MCV, RDW, ferritin, sTfR, and CRP). sTfR and MCHC markers were only available for years 1–5 and years 1–2, respectively. The association between anemia/IDA/ID and adiposity groups was analyzed by logistic regression analyses.

To evaluate how each adiposity measure individually predicts or relates to each iron biomarker, every regression analysis included 1 dependent variable (a hematological marker for linear regression and anemia/ID/IDA for logistic regression), 1 independent variable (an adiposity measure), and several covariates (age, smoking status, educational level, use of antacids or proton pump inhibitors, use of hormonal contraceptives, and intake of iron, heme iron, nonheme iron, and beta-carotene). The same was repeated with all dependent variables. All the *P* values were corrected for multiple comparisons using Benjamini–Hockberg test. Collinearity was assessed with the variance inflation factor, which resulted >5 in all the analyses (low collinearity).

Weighting to account for differences in sample selection and response among the periods of the survey was applied according to NDNS instructions (https://doc.ukdataservice.ac.uk/). A new weighting variable was created by combining the weights from each survey period, taking into account the proportion of participants from each period. This new weighting variable was then used to weight the cases in all analyses.

Statistical analyses were performed using SPSS (Statistical Package for the Social Sciences software, version 29; IBM). Results were considered significant at *P* < 0.05.

## Results

Figure 1 shows the selection process of the participants included. A total of *n* = 1098 women were included in the final analyses, *n* = 496 categorized as normal weight and *n* = 602 categorized by BMI as overweight/obesity. Table 2 shows the general characteristics of the sample population. WRA with overweight/obesity were older (*P* < 0.001) and had lower educational status compared with normal weight WRA (*P* < 0.001). A total of 10.3% of women were taking supplements containing iron, 8.1% in overweight/obesity, and 12.8% in normal weight group (*P* = 0.003). Total iron, heme iron, nonheme iron intake were higher (14.5 compared with 13.8 mg/10 MJ, *P* = 0.002; 0.83 compared with 0.70 mg/10 MJ, *P* < 0.001; 13.6 compared with 13.1 mg/10 MJ, *P* = 0.013, respectively) and beta-carotene intake was lower (*P* = 0.046) in overweight/obesity compared with normal weight WRA. A lower proportion of women with overweight/obesity were using hormonal contraceptives (11.6% compared with 24.2%, *P* < 0.001) and a higher percentage were taking antacids/proton pump inhibitors compared with normal weight women (3.7% compared with 1.3%, *P* = 0.010). Women with overweight/obesity had higher Hb concentrations (132.3 ± 9.5 compared with 130.3 ± 9.2 g/L, *P* < 0.001), red blood cell count (4.4 ± 0.3 compared with 4.3 ± 0.3, 10^12^/L *P* < 0.001), and lower MCH (30.0 ± 2.2 compared with 30.4 ± 2 pg, *P* = 0.002) and MCV (93.0 ± 6.5 compared with 93.8 ± 6.3 fl, *P* = 0.036) compared with normal weight WRA (Table 2). No differences were observed in the remaining iron markers. CRP concentrations were higher in women with overweight/obesity (4.5 ± 6.8 compared with 2.4 ± 4.3 mg/L, *P* < 0.001).

**TABLE 2:** General characteristics of UK women of reproductive age (aged 18–49 y) from NDNS 2008–2019.

	Normal weight (mean/*n*)	SD/%	Overweight/obesity (mean/*n*)	SD/%	*P* value
Age (y)	33.7	9.1	37.0	8.7	<0.001
Current smoker (*n*, %)	134	18.8	162	20.8	0.334
Currently working (*n*, %)	487	68.5	567	73	0.058
Education					
Degree or higher education (*n*, %)	389	54.8	326	42	<0.001
Secondary school (*n*, %)	200	28.2	320	41.2	
Foreign or other (*n*, %)	12	1.7	22	2.8	
Currently full time education (*n*, %)	69	9.7	32	4.1	
No qualifications (*n*, %)	30	4.2	68	8.8	
Country					
England (*n*, %)	607	85.5	650	83.7	0.558
Wales (*n*, %)	25	3.5	45	5.8	
Scotland (*n*, %)	68	9.6	69	8.9	
Northern Ireland (*n*, %)	10	1.4	13	1.7	
Vegan or vegetarian (*n*, %)	33	4.6	45	5.7	0.378
White (*n*, %)	627	88.2	677	87.1	0.537
Anthropometry					
Weight (kg)	60.0	6.2	81.0	13.6	<0.001
BMI (kg/m^2^)	22.2	1.7	30.3	4.8	<0.001
WC (cm)	75.2	6.4	93.1	12.0	<0.001
HC (cm)	97.1	5.6	112.0	9.9	<0.001
WHtR	0.46	0.04	0.57	0.07	<0.001
WHR	0.70	0.05	0.8	0.07	<0.001
Iron markers					
Hemoglobin (g/)L	130.3	9.2	132.3	9.5	<0.001
RBC (10^12^/L)	4.3	0.3	4.4	0.3	<0.001
Ferritin (μg/L)	41.4	44.1	40.9	34.6	0.809
RDW (%)	14.1	1.7	14.2	1.2	0.119
MCH (pg)	30.4	2.0	30.0	2.2	0.002
MCV (fl)	93.8	6.3	93.0	6.5	0.036
MCHC^1^ (g/dL)	33.4	0.8	33.3	0.9	0.328
sTfR^2^ (μg/mL)	4.5	2.5	4.3	1.6	0.188
sTfR-F^2^	3.7	3.5	3.3	2.5	0.098
sTfR/Ferritin^2^	0.31	0.5	0.24	0.4	0.123
CRP (mg/L)	2.4	4.3	4.5	6.8	<0.001
Dietary intake^3^					
4-d food diary complete (*n*, %)	709	99.9	768	98.8	0.016
% misreporting energy needs (%)^4^	−8.1	24.7	−22.9	23.1	<0.001
Energy intake (MJ/d)	6.95	1.78	6.62	1.93	<0.001
Iron dietary, intake (mg/10 MJ)	13.8	3.32	14.5	4.76	0.002
Iron intake including supplements (mg/10 MJ)	16.4	12.9	17.3	22.3	0.360
Heme iron intake (mg/10 MJ)	0.70	0.51	0.83	0.64	<0.001
Nonheme iron intake (mg/10 MJ)	13.1	3.27	13.6	4.62	0.013
Beta-carotene intake μg/10 MJ)	4640.3	5479.9	4144.0	3897.7	0.046
Calcium intake (mg/10 MJ)	1080.6	285.8	1195.1	333.4	0.366
Vitamin C intake (mg/10 MJ)	118.9	75.0	123.0	86.4	0.324
Vitamin A intake (μg/10 MJ)	1343.5	1296.2	1363.3	1823.4	0.810
Alcohol intake (g/d)	8.18	13.8	9.08	16.0	0.248
Medication usage					
Antidepressant drugs (*n*, %)	65	9.2	75	9.9	0.783
Anti-inflammatory drugs (*n*, %)	32	4.5	56	7.4	0.051
Hormonal contraceptive usage (*n*, %)^5^	133	24.2	69	11.6	<0.001
Proton pump inhibitors/antacids (*n*, %)	9	1.3	28	3.7	0.010

Abbreviations: CRP, C-reactive protein; HC, hip circumference; MCH, mean cell hemoglobin; MCHC, mean cell hemoglobin concentration; MCV, mean cell volume; NDNS, National Diet and Nutrition Survey; RDW, red cell distribution width; SD, standard deviation; sTfR, soluble transferrin receptor; sTfR-F, soluble transferrin receptor-ferritin index=sTfR/log10 ferritin; sTfR/Ferritin, soluble transferrin receptor/ferritin; RBC, red blood cells; WC, waist circumference; WHR, waist-to-hip ratio; WHtR, waist-to-height ratio.

Continuous variables are presented as mean and SD. Categorical variables are presented with absolute frequency and percentage. *t* test and *χ*^2^ test were used to assess the difference between normal weight and overweight/obesity as appropriate. *P* < 0.05 considered significant. Weighting applied for all analyses. For ethnicity, age, smoking, education, country, BMI, and diet, “Individual” weight for years 1–11 was applied (weighted sample size: *n* = 1488, Normal weight *n* = 711, Overweight/Obesity *n* = 777); for waist, hip measurements, and medication usage, “Nurse” weight for years 1–11 (weighted sample size: *n* = 1471, Normal weight *n* = 710, Overweight/Obesity *n* = 761) was applied; and for the analysis of iron markers and CRP, “Blood” weight for years 1–11 (weighted sample size *n* = 1148, Normal weight *n* = 559, Overweight/Obesity *n* = 589) was applied.

1 Data available for years 1–2 (weighted sample size *n* = 205, Normal weight *n* = 86, Overweight/Obesity *n* = 119).

2 Data available for years 1–5 (weighted sample size *n* = 563, Normal weight *n* = 273, Overweight/Obesity *n* = 290).

3 Adjusted for percentage of misreporting energy needs.

4 Calculated by Energy intake-Estimated energy requirements (EER)/EER) × 100.

5 Data available for years 1–8 (weighted sample size: *n* = 1146, Normal weight *n* = 549, Overweight/Obesity *n* = 597).

Controlling for age, smoking, educational status, antacids/proton pump inhibitor usage, hormonal contraceptive usage, and total iron/heme iron/nonheme iron/beta-carotene dietary intake, RBC was positively associated with all measures of adiposity (particularly with BMI β: 0.292, *P* < 0.001, and WHtR β: 0.276, *P* < 0.001); Hb was positively associated with BMI, WC, and WHtR (BMI β: 0.130 *P* < 0.001, WC β: 0.098, *P* = 0.009; WHtR β: 0.090, *P* = 0.018, respectively) (Table 3). MCH and MCV were negatively and RDW positively associated with all measures of adiposity (all *P* < 0.001), and MCHC was negatively associated with WHR (β: –0.181, *P* = 0.017). sTfR was positively associated with WC (β: 0.126, *P* = 0.006), WHtR (β: 0.110, *P* = 0.022), and WHR (β: 0.198, *P* < 0.001). No association was found between adiposity measures and ferritin. All adiposity measures were positively associated with CRP (all *P* < 0.001) (Table 3).

**TABLE 3:** Association between iron markers, CRP, and adiposity measures.

		BMI	WC	WHtR	WHR
Hb (g/L)	β	0.130	0.098	0.090	–0.009
	B	0.222	0.071	10.336	–1.256
	*P* value^1^	<0.001	0.009	0.018	0.922
RBC (10^12^/L)	β	0.292	0.271	0.276	0.149
	B	0.017	0.007	1.103	0.716
	*P* value^1^	<0.001	<0.001	<0.001	<0.001
Ferritin (μg/L)^2^	β	0.059	0.049	0.040	0.000
	B	0.009	0.003	0.395	0.000
	*P* value^1^	0.136	0.209	0.320	1.000
RDW (%)^2^	β	0.083	0.123	0.115	0.147
	B	0.001	0.001	0.133	0.203
	*P* value^1^	0.036	0.001	0.004	<0.001
MCH (pg)	β	–0.176	–0.186	–4.985	–0.156
	B	–0.066	–0.030	–0.197	–4.728
	*P* value^1^	<0.001	<0.001	<0.001	<0.001
MCV (fL)	β	–0.164	–0.170	–0.182	–0.118
	B	–0.171	–0.076	–12.82	–9.973
	*P* value^1^	<0.001	<0.001	<0.001	0.001
MCHC (g/dL)^3^	β	0.022	0.016	–0.027	–0.181
	B	0.003	0.001	–0.295	–2.784
	*P* value^1^	0.747	0.227	0.709	0.017
sTfR, (μg*/*mL)^2^^,^^4^	β	0.047	0.126	0.110	0.198
	B	0.003	0.004	0.484	1.078
	*P* value^1^	0.314	0.006	0.022	<0.001
CRP (mg/L)^2^	β	0.427	0.421	0.446	0.274
	B	0.063	0.027	4.476	3.294
	*P* value^1^	<0.001	<0.001	<0.001	<0.001

Abbreviations: CRP, C-reactive protein; Hb, hemoglobin; HC, hip circumference; MCH, mean cell hemoglobin; MCHC, mean cell hemoglobin concentrations; MCV, mean cell volume; RBC, red blood cell count; RDW, red cell distribution width; sTfR, soluble transferrin receptor; WC, waist circumference; WHR, waist-to-hip ratio; WHtR, waist-to-height ratio.

Multiple linear regression analysis adjusted for age, smoking, educational status, antacids/proton pump inhibitors usage, hormonal contraceptive usage, total iron/heme-iron/nonheme iron/beta-carotene dietary intake. *P* < 0.05 considered significant. β standardized coefficient. B unstandardized coefficient. Weighting applied for all analyses.

1 Multiple comparisons corrected *P* value by the Benjamini–Hockberg test.

2 log transformed variables.

3 Data available for years 1–2 (weighted sample size *n* = 205, Normal weight *n* = 86, Overweight/Obesity *n* = 119).

4 Data available for years 1–5 (weighted sample size *n* = 563, Normal weight *n* = 273, Overweight/Obesity *n* = 290).

**FIGURE 2:**
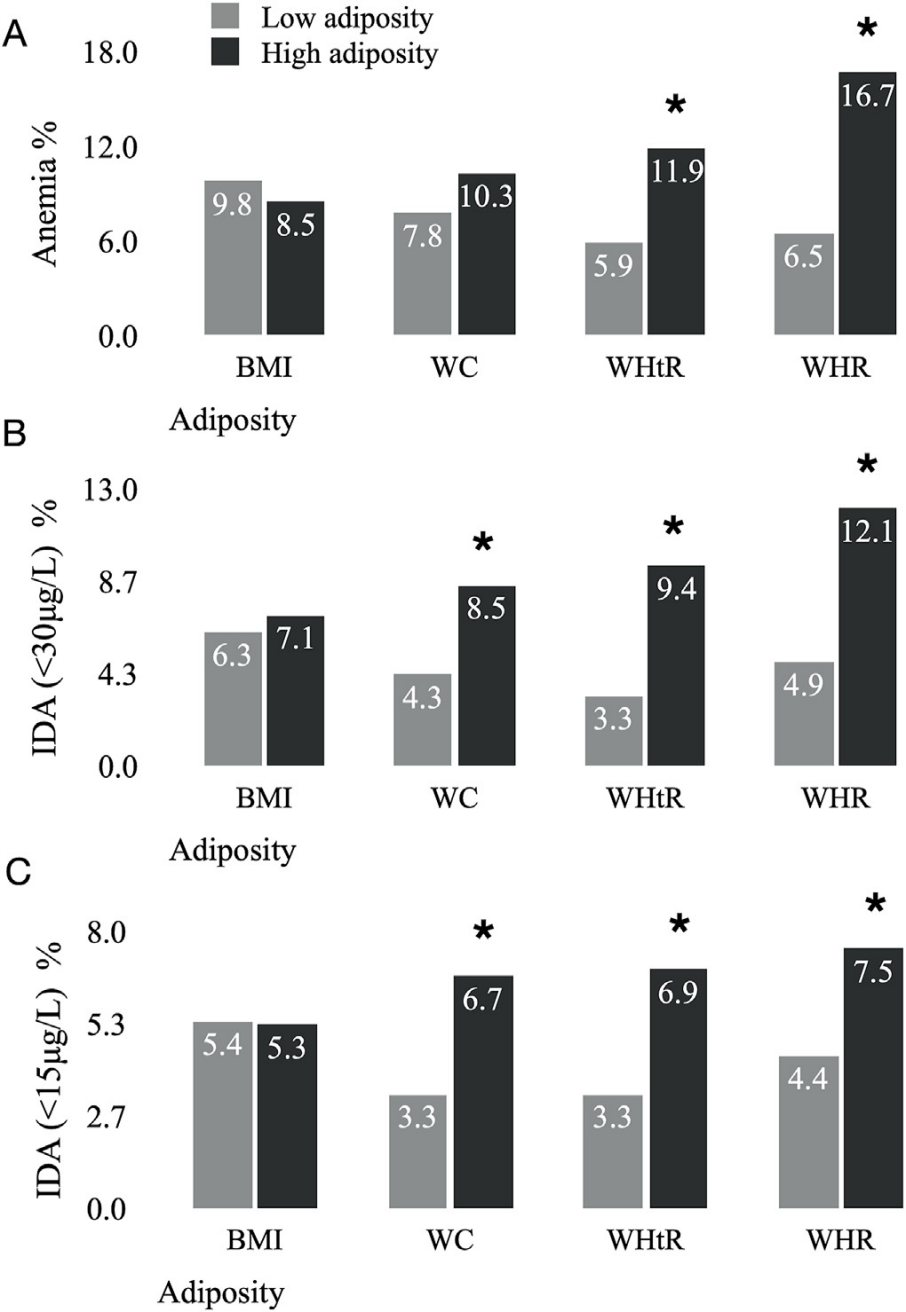
Prevalence of anemia and IDA according to BMI, WC, WHtR, and WHR groups. Data shown as percentages. Weighting applied for all analyses (“Blood” weight for years 1–11, weighted sample size *n* = 1148). *χ*^2^ test was used to assess differences between categories. *P* < 0.05 considered significant. ∗*P* value <0.05. (A) Anemia: hemoglobin <120 g/L. (B) IDA: hemoglobin <120 g/L and ferritin <30 μg/L). (C) IDA: hemoglobin <120 g/L and ferritin <15 μg/L). Adiposity categories: BMI, low adiposity <25 kg/m^2^ (weighted *n* = 559) high adiposity ≥25 kg/m^2^ (weighted *n* = 589); WC, low adiposity <80 cm (weighted *n* = 490), high adiposity ≥80 cm (weighted *n* = 658); WHtR, low adiposity <0.50 (weighted *n* = 511), high adiposity ≥0.50 (weighted *n* = 637); WHR, low adiposity <0.85 (weighted *n* = 842), high adiposity ≥0.85 (weighted *n* = 306). IDA, iron deficiency anemia; without anemia; WC, waist circumference; WHR, waist-to-hip ratio; WHtR, waist-to-height ratio.

**FIGURE 3:**
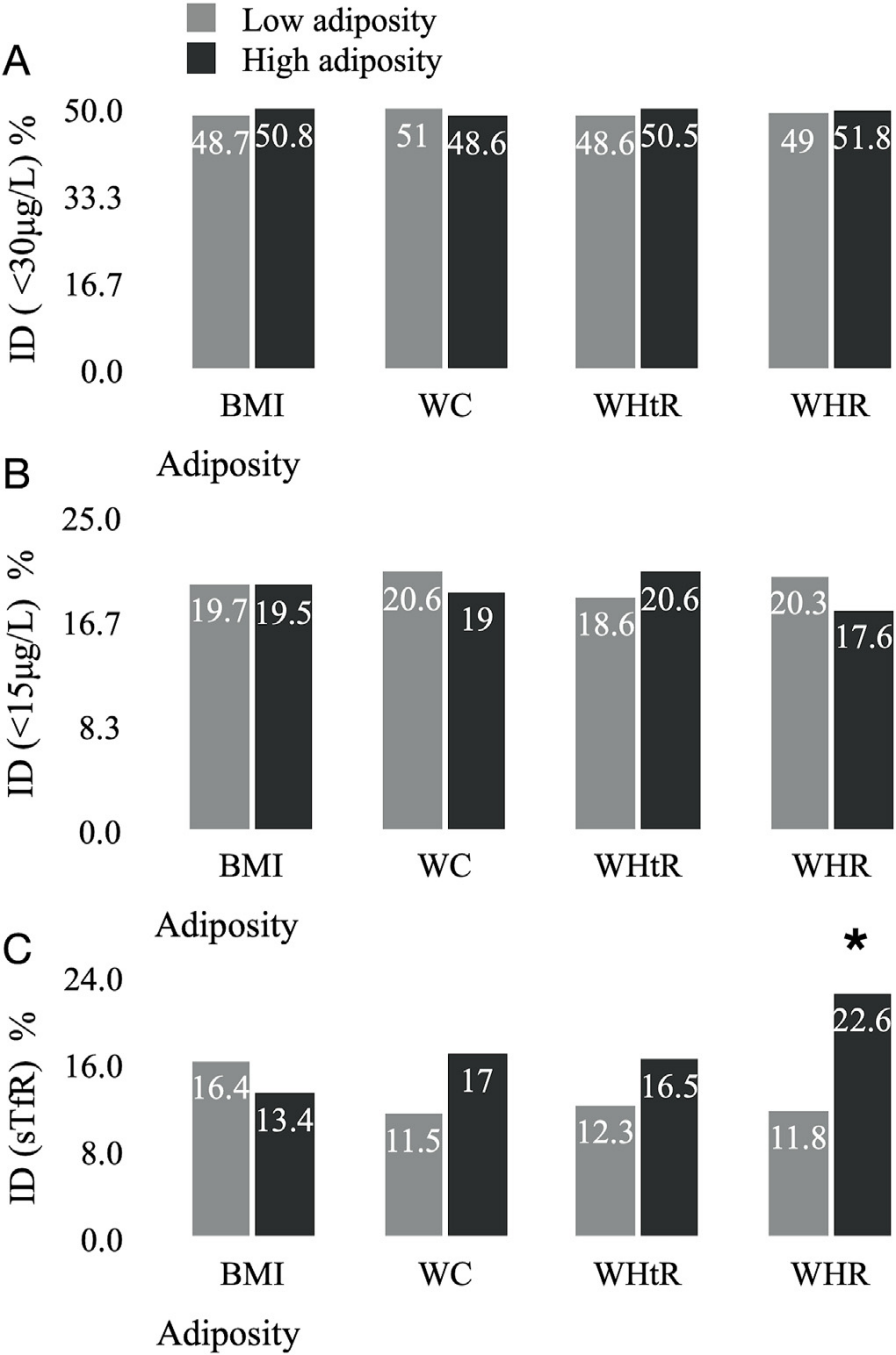
Prevalence of ID defined by ferritin and sTfR concentrations, according to BMI, WC, WHtR, and WHR groups. Data shown as percentages. Weighting applied for all analyses. “Blood” weight for years 1–11, weighted sample size *n* = 1148). *χ*^2^ test was used to assess differences between categories. *P* < 0.05 considered significant. ∗*P* value <0.05. (A) ID defined as ferritin <30 μg/L. (B) ID defined as ferritin <15 μg/L. (C) ID defined as sTfR ≥5.81 mg/L. Adiposity categories: BMI, low adiposity <25 kg/m^2^ high adiposity ≥25kg/m^2^; WC, low adiposity <80 cm, high adiposity ≥80 cm; WHtR, low adiposity <0.50, high adiposity ≥0.50; WHR, low adiposity <0.85, high adiposity ≥0.85. ID, iron deficiency; sTfR, soluble transferrin receptor; WC, waist circumference; WHR, waist-to-hip ratio; WHtR, waist-to-height ratio.

The overall prevalence of anemia in this sample was 9.2%. The prevalence of IDA was 6.8% using the <30 μg/L cutoff, and 5.3% using the <15 μg/L cutoff. The overall prevalence of ID was: 49.7% defined as ferritin <30 μg/L, 19.6% defined as ferritin <15 μg/L, and 6.6% defined as sTfR ≥5.81 mg/L. The proportion of women with anemia was higher in women with WHtR ≥0.50 and WHR ≥0.85 (11.9% compared with 5.9% *P* <0.001; 16.7% compared with 6.5%, *P* < 0.001), with no differences in the anemia prevalence observed between the BMI or WC groups (Figure 2). The prevalence of IDA using both ferritin cutoffs did not differ between BMI groups (*P* = 0.637) (Figure 2). Using either the 15 μg/L or 30 μg/L cutoff, the categories WC ≥80 cm, WHtR ≥0.50, and WHR ≥0.85 had a higher prevalence of IDA compared with those with lower adiposity [IDA (ferritin <30 μg/L): WC 8.5% compared with 4.3%, *P* = 0.008; WHtR: 9.4% compared with 3.3%, *P* < 0.001; and WHR: 12.1% compared with 4.9%, *P* < 0.001; IDA (ferritin <15 μg/L): WC: 6.7% compared with 3.3%, *P* = 0.001; WHtR: 6.9% compared with 3.3%, *P* = 0.007; WHR: 7.5% compared with 4.4%, *P* = 0.036] (Figure 2). The prevalence of ID did not vary between adiposity groups when it was defined with ferritin, both using the cutoff of 30 μg/L and 15 μg/L (Figure 3). When ID was defined as sTfR ≥5.81 mg/L, the prevalence was higher in women with higher WHR (22.6% compared with 11.8%, *P* < 0.001) (Figure 3).

Table 4 shows the association between adiposity measures and anemia, IDA, ID (using ferritin), and ID (using sTfR concentrations). No association was found between BMI/WC and anemia, nor with the different definitions of IDA and ID. Greater adiposity classified by non-BMI measures was associated with anemia and IDA, WHtR ≥0.50 and WHR ≥0.85 being significant predictors of anemia and IDA [anemia: WHtR adjusted odds ratio (aOR) 2.006, *P* = 0.036; WHR aOR 4.489 *P* < 0.001; IDA (ferritin <30 μg/L): WHtR aOR 2.942, *P* = 0.012; WHR aOR 4.142, *P* < 0.001; IDA (ferritin <15 μg/L): WHR aOR 2.778, *P* = 0.004]. Although no association was found between the adiposity groups and ID defined by ferritin, when ID was defined by sTfR, WHR ≥0.85 was a predictor of ID (WHR: aOR 2.104, *P* = 0.004) (Table 4).

**TABLE 4:** Association between adiposity measures and anemia, IDA, ID defined by ferritin concentrations, and sTfR concentrations.

	BMI^1^			WC^2^			WHtR^3^			WHR^4^		
	aOR	CI 95%	*P* value^5^	aOR	CI 95%	*P* value^5^	aOR	CI 95%	*P* value^5^	aOR	CI 95%	*P* value^5^
Anemia^6^	0.653	0.4, 1.0	0.540	1.320	0.8, 2.2	0.436	2.006	1.1, 3.4	0.036	4.489	2.7, 7.4	<0.001
IDA (ferritin <30 μg/L)^7^	0.846	0.4, 1.5	0.673	2.087	1.1, 3.9	0.144	2.942	1.5, 5.8	0.012	4.142	2.3, 7.3	<0.001
IDA (ferritin <15 μg/L)^8^	0.572	0.3, 1.1	0.303	2.248	1.03, 4.8	0.123	1.882	0.9, 3.9	0.196	2.778	1.4, 5.3	0.004
ID (ferritin <30 μg/L)^9^	0.976	0.7, 1.3	0.868	0.942	0.7, 1.2	0.693	1.108	0.8, 1.5	0.601	1.143	0.8, 1.5	0.410
ID (ferritin <15 μg/L)^10^	0.863	0.6, 1.2	0.652	0.894	0.6, 1.3	0.673	1.101	0.7, 1.6	0.623	0.835	0.5, 1.2	0.474
ID (sTfR)^11^	0.783	0.4, 1.2	0.658	1.603	0.9, 2.7	0.436	1.462	0.8, 2.4	0.228	2.104	1.2, 3.4	0.004

Abbreviations: ID, iron deficiency; IDA, iron deficiency anemia; sTfR, soluble transferrin receptor; WC, waist circumference; WHR, waist-to-hip ratio; WHtR, waist-to-height ratio.

Data shown as adjusted odds ratio and 95% CI. Multiple logistic regression adjusted for age, smoking, educational status, antacids/proton pump inhibitors usage, hormonal contraceptive usage, total iron/heme iron/nonheme iron/beta-carotene dietary intake. Anthropometric variables as dichotomous independent variables. Weighting applied for all analyses. “Blood” weight for years 1–11 was applied (weighted sample size *n* = 1148).

*P* < 0.05 considered significant.

1 BMI: Overweight/Obesity (BMI ≥25 kg/m^2^) compared with normal weight (BMI <25 kg/m^2^, reference).

2 WC: ≥80 cm compared with <80 cm (reference).

3 Waist to height ratio: ≥0.50 compared with <0.50 (reference).

4 Waist to hip ratio: ≥0.85 compared with <0.85 (reference).

5 Multiple comparisons corrected *P* value by the Benjamini–Hockberg test.

6 hemoglobin <120 g/L.

7 hemoglobin <120 g/L and ferritin <30 μg/L.

8 hemoglobin <120 g/L and ferritin <15 μg/L.

9 ferritin <30 μg/L.

10 ferritin <15 μg/L.

11 sTfR ≥5.81 mg/L.

## Discussion

In this study, the association between various measures of adiposity and the indicators of iron status in UKWRA was assessed. WRA with higher central adiposity, measured by WC, WHtR, and WHR, had higher odds of developing anemia and IDA compared with those with lower central adiposity. Even after adjusting for inflammation, ID defined by ferritin concentrations did not show any association with adiposity in any categorization. However, when sTfR was used to define ID, it was significantly associated with central obesity.

Our findings have shown that despite having higher dietary iron intake, WRA with increased adiposity exhibited an impaired iron profile compared with those with lower adiposity. Contrary to our hypothesis and supported by other evidence [16,19,20,31,[48], [49], [50], [51], [52], [53]], adiposity measures were positively associated with Hb in the linear regression analysis. However, other iron markers such as MCV, MCH, RDW, MCHC, and sTfR showed a trend toward impaired iron status. Cepeda-Lopez et al. [17] showed that WRA with obesity had higher odds of having ID [defined as serum iron <60 μg/dL or total iron binding capacity (TIBC) >360 μg/dL or TSAT <20%] compared with normal weight WRA [aOR: 1.92; 95% confidence interval (CI): 1.23, 3.01]. Yanoff et al. [16] defined ID as sTfR >27.5 nmol/L and reported that adults with obesity had higher odds of developing ID than adults without obesity (OR: 1.8; 95% CI: 1.1, 3, *P* = 0.040); but when they used ferritin for the same analysis, they did not find differences (OR: 0.9, *P* = 0.710). Herter-Aeberli et al. [20] reported a positive association between overweight/obesity and sTfR (BMI ≥23 kg/m^2^). Choma et al. [31] reported that WRA with overweight and obesity were more likely to have ID (defined as ferritin <15 μg/L or low TSAT/serum iron when CRP >12 mg/L), but this study also showed a lower risk of ID when WC ≥80 cm was considered in the analysis. Jordaan et al. [54] described that BMI, WC, and body fat percentage were negatively associated with MCV, MCH, and TSAT in WRA (*P* < 0.001), not observing differences in ferritin concentrations between adiposity groups. Kerkadi et al. [19] evaluated adults aged 20–50 y with no differences in the prevalence of anemia between WC groups but reported an inverse association between BMI/WC and serum iron/TSAT. Laudisio et al. [55] studied young individuals with obesity classes II and III and found no association between adiposity and serum iron when BMI was considered; however, and similar to our findings, WC and % fat mass were negatively associated with serum iron, and WC was the best predictor of serum iron. In a case-control study, Nassar et al. [56] reported significantly lower serum iron in obesity compared with normal weight (38.9 ± 29.7 compared with 53.3 ± 26.5 mg/dL). They also showed that BMI correlated positively with TIBC, negatively with serum iron, and negatively associated with Hb. Stoffel et al. [30] reported that the ratio of android fat/total fat measured by DEXA in WRA positively correlated with higher hepcidin, TIBC, CRP/α1-acid glycoprotein, and negatively with TSAT. Evidence from NHANES survey [57] also found that women with obesity classified with BMI had a higher sTfR than those who were overweight and normal weight (*P* < 0.0167). Most of these studies have shown iron status impairment using markers other than ferritin, and mostly using BMI as a marker of adiposity. The current research confirms not only a connection between increased adiposity and compromised iron status as measured by markers other than ferritin such as sTfR but also that increased central adiposity is a risk factor for ID, particularly for progressing to its most severe stage, IDA.

Regardless of adiposity, the prevalence of ID varied significantly between the different definitions (ferritin <15 μg/L, ferritin <30 μg/L, and sTfR > 5.81 mg/L). However, this study reveals that ID is highly prevalent in UK WRA, particularly when the threshold of ferritin <30 μg/L is applied. Considering a ferritin cutoff of 30 μg/L for ID as defined in NICE guidelines [32] and recently proposed for ID screening in this population [58], the overall prevalence of ID in this analysis was almost 50%. An Irish study reported an overall ID prevalence of 37% in adults using unadjusted ferritin [59]. Unlike some research, including the aforementioned Irish study and the present one, which used a 30 μg/L cutoff, most studies reporting ID prevalence follow WHO guidelines and define it as ferritin <15 μg/L. This is the case in most European research, where ID prevalence ranges between 3% and 32% [60]. Considering this cutoff, the prevalence in our study was similar to those reported in European countries [60]. Even using the ferritin cutoff of 15 μg/L, at least 1 in 5 WRA from this representative sample were iron deficient, with WRA with overweight/obesity being the most vulnerable given their higher prevalence of anemia, IDA, and ID.

Supporting our hypothesis, Wang et al. [61] conducted a Mendelian randomization study, using single nucleotide polymorphisms associated with obesity and genetic variants associated with IDA in adults aged 40–69 y. An inverse variance-weighted association was found between IDA and WC, trunk fat mass, trunk fat percentage, and body fat percentage (OR: 1.003–1.004, *P* < 0.001). Mendelian randomization is more reliable than observational studies for testing causal associations because it is less vulnerable to confounding or reverse causality [62]. Therefore, the findings of Wang et al. [61] reinforce our hypothesis that adiposity is associated with the development of IDA, further adding causality to this association.

Unlike the previously mentioned studies, other studies have shown opposite results. An inverse or no association between BMI and anemia/IDA has been reported [[48], [49], [50],[63], [64], [65], [66], [67]] or between BMI and ID [48,[51], [52], [53],^66^]. Most of these studies have included participants with underweight [[49], [50], [51], [52],[63], [64], [65]], which may mask any relationship between BMI and anemia. Additionally, in the study by Fanou-Fogny et al. [52], only 8.7% of WRA had obesity defined by BMI, and no participants with obesity had anemia or ID (defined by ferritin <12 μg/L and sTfR >8.3 mg/L). Suarez-Oregon et al. [53] found a protective effect of all measures of adiposity (BMI, WC, and WHtR) on ID, when defined ferritin <15 μg/L, <30 μg/L, and on anemia. The percentage of obesity in the latter study was 17.3% in contrast to our study, which was 21.7%. In many of these studies, Hb was the sole iron marker assessed [49,50,64] and in the studies that ferritin was evaluated, it was not adjusted for inflammation [48,51,52,66,67]. The ferritin adjustment could have had particular importance in the study from Arshad et al. [67], which included adults with BMI >40 kg/m^2^, a group that is highly likely to have elevated inflammation. Suarez-Oregon et al. [53] performed the ferritin adjustment by adding CRP to the regression model, which may not have the same results as adjusting the concentration of each individual value using CRP, as previously described [35,44,45]. In addition, in 3 of these studies, participants with high CRP and ferritin were excluded, which could have selected the population at a lower risk of IDA [48,52,66].

Iron metabolism impairment in women with greater adiposity may be linked to chronic inflammation. Adipose tissue, an endocrine organ, secretes adipokines and cytokines systemically. In obesity, this tissue becomes hypertrophic and hyperplastic, with activated macrophages that reduce anti-inflammatory cytokines and produce proinflammatory mediators locally and systemically [68,69]. In addition to CRP, increased nitric oxide, tumor necrosis factor alpha (TNF-α), and IL-6 have been reported in women with overweight/obesity [[70], [71], [72]]. IL-6 stimulates hepcidin production/secretion [69], the main regulator of iron metabolism, which could reduce iron absorption and availability for erythropoiesis [12,30,73], altering the hematological markers MCV, MCH, and RDW. Reactive oxygen species and nitric oxide could favor less deformability of red blood cells, increasing their clearance and reducing their circulating number [74]. TNF-α can increase endothelial adhesion to red blood cells and accelerate red blood cell clearance [75] and reduce the response of the bone marrow to the hormone erythropoietin (EPO) in its attempt to stimulate the production of red blood cells [76]. This could generate a greater demand for EPO to maintain a normal Hb; therefore, the EPO concentration would be elevated chronically [77]. EPO production in individuals with inflammation would fail at a later stage with Hb concentration decreasing, resulting in anemia [77]. The increase in EPO concentration could explain the slightly higher mean Hb and RBC in the overweight/obesity group and their positive linear association with all measures of adiposity in this study.

Considering the above, despite having a greater dietary iron intake compared with normal weight WRA, we propose that WRA with higher central adiposity have an impaired iron status, possibly mediated by a chronic proinflammatory state that would favor lower iron availability for erythropoiesis, increased clearance of circulating red blood cells, and less effective erythropoiesis that would result in a higher prevalence of ID and particularly IDA. Future studies are necessary to better understand these mechanisms and evaluate the influence of adiposity on iron status in pregnancy and its possible adverse consequences.

This study is limited by its observational design and the absence of additional markers of iron status, such as serum iron, TSAT, hepcidin, and EPO, which would have been valuable for this analysis. Another limitation is the lack of chronic inflammation markers, such as α1-acid glycoprotein, which would have allowed a more accurate and precise estimation of ferritin concentrations. Additionally, blood loss or heavy menstrual periods information was not available for this analysis. However, this study used nationally representative data, including a large sample size with well-characterized anthropometric measurements including central adiposity measures and available and complete data on iron intake. Ferritin was also corrected for inflammation, enabling an accurate assessment of the IDA/ID diagnoses.

This study demonstrates that at least 1 in 5 WRA in the UK are iron deficient, highlighting that current policies may be ineffective in detecting and managing this deficiency in this vulnerable population group. Moreover, greater central adiposity is a risk factor for the development of ID and IDA in UK WRA, highlighting the importance of central adiposity measures as better predictors of ID/IDA. This study also reaffirms the urgent need to address overweight/obesity and ID as a combined public health problem, given the significant and increasing prevalence in WRA.

## Author contributions

The authors’ responsibilities were as follows – MTM, MAK, MSM, PDT, ML, SPD: designed research; SPD: conducted research and analyzed data; SPD and MTM: wrote the article; MTM: had primary responsibility for final content; and all authors: read and approved the final manuscript.

## Conflict of interest

MTM reports a relationship with Solvotrin Therapeutics that includes funding grants. ML reports a relationship with Solvotrin Therapeutics that includes board membership, equity or stocks, and funding grants. ML has patent #WO2017158030A1 Compositions and methods for increasing iron intake in a mammal issued to Trinity College Dublin and Solvotrin Therapeutics. All other authors report no conflicts of interest.

## Funding

No specific funds required for the completion of this research. SPD is a PhD Student funded by Department for the Economy scholarship, Ulster University.

## Data availability

Data described in the manuscript, code book, and analytic code will be made available upon request pending.
